# Supplementary material for: The Systems Biology Research Tool: evolvable open-source software
Source: BMC Syst Biol. 2008 Jun 29;2:55. doi: 10.1186/1752-0509-2-55 (PMC2446383; doi:10.1186/1752-0509-2-55)
Supplement: Additional file 1 — SBRT Archive. An archive of the current version of the Systems Biology Research Tool. [file 1752-0509-2-55-S1.zip › sbrt-1.4.0/doc/developers_guide/api/index-files/index-21.html]

U-Index


|  |  |  |
| --- | --- | --- |
| **Overview** \| Package \| Class \| Use \| **Tree** \| **Deprecated** \| **Index** \| **Help** | |  |
| **PREV LETTER**   **NEXT LETTER** | **FRAMES**    **NO FRAMES**     **All Classes** |  |


A B C D E F G H I J K L M N O P Q R S T U V W X Y Z 

---


## **U**

**UnboundedVariableException** - Exception in sbrt.kernel.math: This class is used to indicate that a variable is unbounded. **UnboundedVariableException(Object)** - Constructor for exception sbrt.kernel.math.UnboundedVariableException: Constructs a new exception from the provided unbounded variable. **unchangedFreeVariables()** - Method in class sbrt.kernel.fba.comp.CdHarFluxVectorComputer.Results: Returns the number of free variables whose values are the same as they were in the initial flux vector. **unchangedVariables()** - Method in class sbrt.kernel.math.geom.comp.CdHarComputer.Results: Returns the number of variables whose values are the same as they were in the previous point. **UniqueCycleIdManager** - Class in sbrt.shell.mng.graph\_theory: This class is used to identify the unique cycles in files that contain collections of cycles. **UniqueCycleIdManager()** - Constructor for class sbrt.shell.mng.graph\_theory.UniqueCycleIdManager: **unlock(Object)** - Static method in class sbrt.ext.R.R: Unlocks the R engine so that others have access, that is, someone can request a new key. **UnmodifiableCollection**<E> - Class in sbrt.kernel.util: This class is used to create an unmodifiable view of collections. **UnmodifiableCollection(Collection<E>)** - Constructor for class sbrt.kernel.util.UnmodifiableCollection: Constructs a new unmodifiable view of the provided collection. **UnmodifiableIterator**<E> - Class in sbrt.kernel.util: This class is used to create an iterator that cannot modify its underlying collection. **UnmodifiableIterator(Iterator<E>)** - Constructor for class sbrt.kernel.util.UnmodifiableIterator: Constructs a new unmodifiable iterator from the provided iterator. **UnmodifiableList**<E> - Class in sbrt.kernel.util: This class is used to create an unmodifiable view of lists. **UnmodifiableList(List<E>)** - Constructor for class sbrt.kernel.util.UnmodifiableList: Constructs a new unmodifiable view of the provided list. **UnmodifiableMap**<K,V> - Class in sbrt.kernel.util: This class is used to create an unmodifiable view of maps. **UnmodifiableMap(Map<? extends K, ? extends V>)** - Constructor for class sbrt.kernel.util.UnmodifiableMap: Constructs a new unmodifiable view of the provided map. **UnmodifiableSet**<E> - Class in sbrt.kernel.util: This class is used to create an unmodifiable view of sets. **UnmodifiableSet(Set<E>)** - Constructor for class sbrt.kernel.util.UnmodifiableSet: Constructs a new unmodifiable view of the provided set. **upperBoundsEqualZero(Map<?, Interval>, double)** - Method in class sbrt.kernel.fba.analysis.FluxIntervalAnalyzer: Returns the number of provided intervals whose upper bounds equal zero. **upperBoundsEqualZero(Collection<Interval>, double)** - Method in class sbrt.kernel.fba.analysis.FluxIntervalAnalyzer: Returns the number of provided intervals whose upper bounds equal zero. **upperBoundsGreaterThanZero(Map<?, Interval>, double)** - Method in class sbrt.kernel.fba.analysis.FluxIntervalAnalyzer: Returns the number of provided intervals whose upper bounds are greater than zero. **upperBoundsGreaterThanZero(Collection<Interval>, double)** - Method in class sbrt.kernel.fba.analysis.FluxIntervalAnalyzer: Returns the number of provided intervals whose upper bounds are greater than zero. **USAGE** - Static variable in class sbrt.Sbrt: Indicates the typical way in which this program is called from the command line. **Util** - Class in sbrt.kernel.util: This class contains static methods that perform utility functions.

---


|  |  |  |
| --- | --- | --- |
| **Overview** \| Package \| Class \| Use \| **Tree** \| **Deprecated** \| **Index** \| **Help** | |  |
| **PREV LETTER**   **NEXT LETTER** | **FRAMES**    **NO FRAMES**     **All Classes** |  |


A B C D E F G H I J K L M N O P Q R S T U V W X Y Z 

---
